# Incidence of severe immune-related adverse reactions in patients with HIV and cancer receiving immune checkpoint inhibitors: a systematic review and meta-analysis

**DOI:** 10.3389/fonc.2026.1741760

**Published:** 2026-03-17

**Authors:** Danyan Zhang, Xinlong Zhang, Xuan Liu, Ruobao Li, Yan Huang

**Affiliations:** 1 Affiliated Hospital of Shandong Second Medical University, School of Clinical Medicine, Shandong Second Medical University, Weifang, Shandong, China; 2 Jinming Yu Academician Workstation of Oncology, Shandong Second Medical University, Weifang, Shandong, China; 3 Department of Human Anatomy, Shandong Second Medical University, Weifang, Shandong, China

**Keywords:** cancer, HIV-infected, immune checkpoint inhibitors, immune-related adverse event - irAE, safety and outcomes

## Abstract

**Background:**

With the continuous advancement of various treatment modalities, such as antiretroviral therapy, the life expectancy of people living with HIV (PLWH) is approaching that of the general population. Consequently, the annual incidence of non-AIDS-defining malignancies in this population is increasing. Immune checkpoint inhibitors (ICIs) block T cell suppression, enabling T cells to kill tumor cells. They also restore the function of HIV-specific CD8+ T cells and enhance their ability to clear cells with latent HIV infection. However, it is unclear if this will create an immune imbalance in the PLWH population, thereby increasing the likelihood of more severe immune-related adverse events, which is a common concern. Thus, this study aimed to assess the incidence of serious immune-related adverse events associated with ICI use in PLWH with cancers. This will provide clinicians with reliable evidence to optimize therapeutic decisions and ensure patient safety.

**Methods:**

A thorough systematic search of three major databases (PubMed, Embase, and Web of Science) was conducted from database inception to July 13, 2025. We extracted the necessary data and their corresponding 95% confidence intervals (CIs) for use in the subsequent meta-analysis. Statistical analyses were performed using the R software (version 4.5.1).

**Results:**

This meta-analysis included 18 studies involving 813 patients. The final results indicated that without considering CD4+ T cell counts, the incidence rate of severe immune-related adverse reactions was 9% (95% CI: 0.08–0.12). The rate is relatively close to the spectrum of irAE incidence observed with ICI therapy in HIV-uninfected cancer patients. We further stratified CD4+ T cell counts by extracting data from seven studies. The findings showed that the incidence of severe immune-related adverse events was 13% (95% CI: 0.08-0.21) in those with a CD4+ T cell count <200 cells/μL, versus 11% (95% CI: 0.09-0.15) in those with ≥200 cells/μL. There was no statistically significant difference between the two groups (p=0.4444).

**Conclusion:**

Preliminary data from this study show that the pooled incidence of severe irAEs induced by ICIs is approximately 9% in ART-treated and clinically stable PLWH with cancer. The rate is relatively close to the spectrum of irAE incidence observed with ICI therapy in HIV-uninfected cancer patients. Furthermore, subgroup analysis based on CD4+ T cell count revealed no statistically significant difference in the incidence of severe irAEs between the groups. Therefore, current evidence does not indicate that baseline CD4+ T cell count is an absolute contraindication to ICI therapy in PLWH with cancer. Taken together, these findings provide preliminary support for an acceptable safety profile of ICIs in PLWH with cancer. Combined with observed preliminary antitumor activity, the results justify further prospective studies. However, the wider confidence interval for the incidence in the subgroup with CD4+ T cell count <200 suggests greater uncertainty in this population, warranting closer monitoring in clinical practice.

## 1 Introduction

Human immunodeficiency virus/acquired immunodeficiency syndrome (HIV/AIDS) infection is a major threat to public health globally. Cervical cancer, Kaposi’s sarcoma, and non-Hodgkin lymphoma are the most common tumors among people living with HIV (PLWH), and they are collectively known as traditional AIDS-defining malignancies (1). The development of these tumors is essentially a direct consequence of the body’s loss of immune control over specific oncogenic viruses due to severe immunodeficiency (2). Non-AIDS-defining cancers are a byproduct of chronic immune dysregulation (3). Even among individuals receiving effective antiretroviral therapy with well-restored CD4 counts, their risk of developing these cancers remains significantly higher than that of the HIV-uninfected individuals. This suggests that their driving factors are more complex. Beyond immunodeficiency, persistent chronic immune activation, systemic inflammation, age-related biological changes, and behavioral risk factors (such as smoking and persistent human papillomavirus (HPV) infection) collectively create the primary oncogenic milieu (1). Based on their relationship with infectious agents, non-AIDS-defining cancers can be further categorized into two groups. The first group consists of virus- (or pathogen-) associated cancers (4). These include liver cancer caused by the hepatitis B virus (HBV); vulvar, anal, and oropharyngeal cancers caused by high-risk HPV; and Hodgkin lymphoma associated with the Epstein-Barr virus (EBV). The second group comprises cancers unrelated to viruses, whose occurrence is not directly linked to a clear infectious agent but is primarily attributed to the chronic inflammation, environmental, and behavioral factors, such as lung cancer and cutaneous melanoma. With continuous advances in treatment modalities, such as antiretroviral therapy, the life expectancy of PLWH has now approached that of the general population, and the incidence of AIDS-defining malignancies has been declining year by year (5). However, this has been accompanied by a marked increase in the incidence of non-AIDS-defining malignancies and is currently one of the primary factors affecting the lifespan of this population (6).

In response to the increasing clinical needs of this patient group, the advent of immune checkpoint inhibitors (ICIs), including those targeting programmed cell death protein-1 (PD-1)/programmed death-ligand-1 (PD-L1) and cytotoxic T-lymphocyte-associated protein 4 (CTLA-4), has led to renewed interest. ICIs hold promise for reactivating latent CD4+ T cells to generate HIV-specific CD8+ T cells, thereby enhancing immune control of HIV (7). Furthermore, ICIs have achieved considerable success in antitumor therapy by blocking inhibitory checkpoints on T cells, thereby reactivating the adaptive antitumor immunity (8). However, a critical clinical question arises: while activating the immune system, do ICIs disrupt the immune equilibrium established during HIV infection? There is widespread concern that ICI use in PLWH may lead to a higher incidence of immune-related adverse events (irAEs). Consequently, PLWH are generally excluded from clinical trials that evaluate the safety and efficacy of ICIs in cancer treatment.

Despite the 2020 ASCO-supported FDA consensus guidelines recommending the inclusion of PLWH with adequate immune function in cancer trials (9), only few trials have conditionally enrolled PLWH (9). Consequently, high-level evidence on ICI use in this population is lacking. Clinical decision-making relies primarily on small case series and observational studies. These fragmented data have yielded inconsistent results, with some demonstrating manageable safety profiles and favorable antitumor activity. However, the precise safety spectrum and risk levels of ICIs remain unclear.

Therefore, we conducted a systematic review and meta-analysis to comprehensively and accurately evaluate the safety of ICIs for PLWH with cancer. This study aimed to provide clinicians with reliable evidence to optimize treatment decisions and safeguard patient safety.

## 2 Materials and methods

This study was conducted according to the recommendations of the Preferred Reporting Items for Systematic Reviews and Meta-Analyses (PRISMA) (10) and was registered in PROSPERO (registration number CRD420251137955).

### 2.1 Search strategy

We systematically searched databases (PubMed, Embase, and Web of Science) using a combination of search terms and free-text phrases to evaluate the safety of ICIs in patients with cancer and HIV infection. The search included articles published from the inception of the databases until July 13, 2025. The search strategy used for each database is shown in Additional File 1.

### 2.2 Inclusion and exclusion criteria

The inclusion criteria were as follows: (1) observational studies (prospective or retrospective cohort studies, case series) and clinical trials (randomized or non-randomized); (2) adult (≥18 years) cancer patients with laboratory-confirmed HIV infection (via PCR or antibody testing), regardless of CD4+ T cell count or HIV viral load; (3) receiving ART therapy with stable HIV disease; (4) use of ICI monotherapy or combination therapy; and (5) extractable irAE incidence data.

The exclusion criteria were as follows: (1) studies without an outcome of interest, (2) patients not diagnosed with tumors (no definitive pathology) or HIV, (3) patients not treated with ICI therapy, (4) unpublished results with incomplete patient characteristics, (5) opinion articles, (6) technical reports, (7) case reports, (8) animal studies, and (9) reviews.

### 2.3 Data extraction and quality assessment

Following literature retrieval, the results were exported to the reference management software EndNote 21, and duplicate studies were excluded. Subsequently, two independent reviewers, DYZ and XLZ, screened the titles and abstracts and assessed any potential full-text materials. Studies that met the eligibility criteria were included in this review.

We conducted a qualitative assessment of the included studies using the Newcastle-Ottawa Scale (NOS). This assessment was performed independently by two researchers (DYZ and XLZ). The NOS comprises three domains, each scored from 0 to 9. In this study, all screened articles received quality assessment scores of six or higher.

Any discrepancies encountered during data extraction and quality assessment were resolved through discussions with the senior author (YH).

### 2.4 Statistical methods

We extracted the total number of participants and the number experiencing grade 3 or higher immune adverse reactions from eligible studies. The extracted data were imported into R software (version 4.5.1) and analyzed using packages such as meta and dmetar. To assess heterogeneity, we employed the Q test and I² value. When I² > 50% or P < 0.1, substantial heterogeneity between studies was considered, permitting the use of a random-effects model. Otherwise, a fixed-effects model was employed. Sensitivity analyses assessed the robustness and reliability of results. Funnel plots and Egger’s test were utilized to analyze publication bias.

Grade 3 or higher irAEs (severe irAEs) were defined according to the following two criteria. First, the event must have been explicitly reported in the original study as an irAE or a suspected immune-mediated adverse reaction, or it was unanimously deemed by two independent reviewers in this study as highly likely to have an autoimmune or autoinflammatory pathological mechanism. This latter determination was based on its clinical presentation (e.g., colitis, pneumonitis, hepatitis, endocrinopathy, rash, etc.) and its timing of onset (typically occurring within weeks to months following ICI administration). Second, to assess severity, we employed the Common Terminology Criteria for Adverse Events (CTCAE), widely used by the U.S. National Cancer Institute, which categorizes toxicities into five grades of increasing severity: asymptomatic/mild (Grade 1), moderate (Grade 2), severe (Grade 3), life-threatening (Grade 4), and death (Grade 5). Severe irAEs were defined as those of Grade 3 or higher (≥3).

For any serious adverse event in the original reports that was not explicitly classified as an irAE, a conservative approach was applied. Such events were not counted as severe irAEs unless compelling supporting evidence was available. This evidence included explicit attribution by the original authors, the presence of specific autoantibodies, or significant clinical improvement following ICI discontinuation and immunosuppressive therapy.

## 3 Results

### 3.1 Search results

Initially, we retrieved 1,127 articles, including 328 from PubMed, 507 from Embase, and 292 from Web of Science. We excluded 479 duplicate records using EndNote 21 software. After screening titles and abstracts, we excluded 616 articles that were deemed irrelevant or failed to meet the inclusion criteria. We conducted a comprehensive full-text review of the remaining 32 articles and excluded 14 studies (12 conference abstracts, one case report, and one review article).

**Figure 1:**
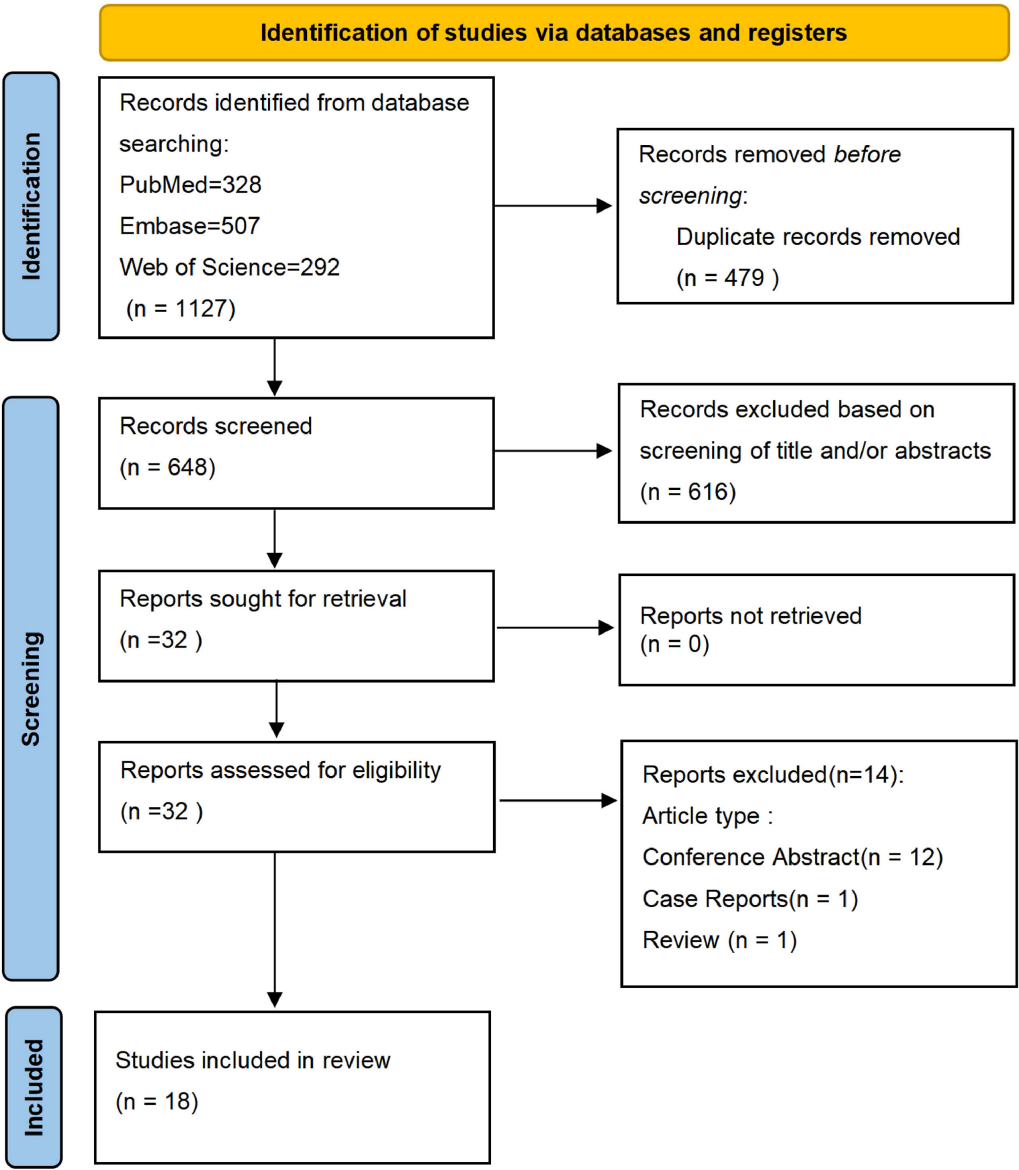
PRISMA flow diagram depicting the article selection and screening process.

The final study included 18 articles with 813 patients who met the inclusion criteria. The literature screening process is illustrated in **Figure 1**.

### 3.2 Characteristics of the included studies

Among the included studies, 12 were retrospective cohort studies and six were prospective cohort studies. These studies were published between 2018 and 2024. The average age of participants in most trials was less than 60 years, and the majority were male. The detailed study characteristics are presented in **Table 1**. Based on the NOS scores, five, three, and ten studies received scores of 8, 7, and 6, respectively (Additional File 2).

**Table 1:** Baseline characteristics of the included studies.

Refs.	Year of publication	Study design	Country	Age	Sex		Patients (N1)	Grade 3–5 AEs (n1)	Grade 3–5 AEs (n2)/Patients (N2)		Tumour type (n3)	ICI Type ± combination therapy (n4)
					Male	Female			CD4+ t cell count < 200 cells/μL	CD4+ t cell count ≥200 cells/μL		
Lambert Assoumou (27)	2024	Prospective cohort study	France	59 (54-64)	111	29	140	20	7/29	11/92	LC (65), head and neck cancer (15), melanoma (12), liver cancer (11), HL (9)	Pembrolizumab (58), nivolumab (49), cemiplimab (4), atezolizumab (15), durvalumab (10), nivolumab +ipilimumab (3), atezolizumab +bevacizumab (1)
Talal El Zarif (24)	2023	Retrospective study	The United States, Europe, and Australia	58 (51-63)	331	59	390	30	5/64	15/152	NSCLC (111), HCC (44), HNSCC (39), anal cancer (28), melanoma (26), SCLC (24), KS (21), HL (15), NHL (15), nonmelanoma skin cancer (13), UC (12), RCC (9), other (33)	Anti-PD-1/anti-PD-L1 monotherapy (274), Anti-PD-1/anti-PD-L1+chemotherapy (68), Anti-PD-1/anti-PD-L1+targeted agents (25), Anti-PD-1+anti-CTLA-4 (23)
Kathryn Lurain (25)	2024	Retrospective study	The United States	48 (26-68)	20	3	23	3	–	–	HL (23)	Nivolumab or pembrolizumab (17), nivolumab + brentuximab vedotin (5), nivolumab + ifosfamide, carboplatin, etoposide (1)
Luling Wu (26)	2023	Retrospective study	China	44 (29-69)	15	0	15	1	1/5	0/1	NHL (7), HL (2), LC (5), NPC (1)	Sintilimab (3), sintilimab +chemotherapy (5), sintilimab +targeted agents (1), sintilimab +chemotherapy +targeted agents (3), nivolumab (1), camrelizumab +chemotherapy (2)
Menghua Wu (28)	2023	Retrospective study	China	58.7 (51.6-65.8)	8	2	10	2	–	–	BC (10)	Camrelizumab (10)
Menghua Wu (29)	2023	Retrospective study	China	59 (41-75)	21	3	24	1	–	–	Seminoma (1), GC (2), renal carcinoma (4), rectal carcinoma (1), NHL (2), anal cancer (1), cervical cancer (1), HCC (2), renal pelvic cancer (1), penile carcinoma (1), BC (6), bile duct cancer (1), ureteral cancer (1)	Camrelizumab (18), camrelizumab +chemotherapy (6)
Yu Xiong (30)	2023	Retrospective study	China	46.5 (29-78)	12	4	16	3	–	–	diffuse large B cell lymphoma (1), HL (3), HCC (3), invasive cervical cancer (3), NSCLC (2), SCLC (1), gastric adenocarcinoma (1), laryngeal cancer (1), anal cancer (1)	Camrelizumab (6), camrelizumab +chemotherapy (3), camrelizumab +targeted agents (6), camrelizumab +chemotherapy +targeted agents (1)
Shahla Bari (31)	2019	Retrospective study	The United States	54 (40-62)	14	3	17	1	0/2	1/14	LC (10), HCC (2), anal cancer (2), kidney cancer (1), NHL (1), basal cell carcinoma (1)	Nivolumab (13), pembrolizumab (3), atezolizumab (1)
Natalie Galanina (32)	2019	Retrospective study	The United States	44 (33-63)	9	0	9	0	–	–	KS (9)	Nivolumab (8), pembrolizumab (1)
Maria Gonzalez-Cao (33)	2020	Prospective cohort study	Spain	54 (30-73)	16	4	20	0	–	–	NSCLC (14), melanoma (2), SCLC (1), anal carcinoma (2), BC (1)	Durvalumab (20)
Lakshmi Rajdev (34)	2023	Prospective cohort study	the United States and Australia	54.6 (47.5-59.3)	33	3	36	5	2/8	3/28	KS (15), anal cancer (4), HNSCC (2), liver cancer (1), NSCLC (2), others (pancreatic cancer, skin squamous cell cancer, breast cancer, gallbladder cancer, follicular dendritic sarcoma, SCLC, colon cancer, squamous cell cancer of unknown primary, liposarcoma, and ovarian cancer) (12)	Nivolumab (36)
Thomas S Uldrick (35)	2019	Prospective cohort study	the United States	57 (39-77)	28	2	30	2	–	–	KS (6), NHL (5), anal cancer (6), squamous cell carcinoma of the skin (3), adenoid cystic carcinoma (1), BC (1), cholangiocarcinoma (1), HCC (1), NSCLC (1), pancreatic cancer (1), papillary urothelial carcinoma (1), prostate cancer (1), sarcomatoid lung carcinoma (1), tonsillar carcinoma (1)	Pembrolizumab (30)
Lorena Ostios-Garcia (36)	2018	Retrospective study	the United States	51 (43-59)	6	1	7	0	0/1	0/5	NSCLC (7)	Pembrolizumab (5), nivolumab (2)
Kathryn Lurain (37)	2021	Retrospective study	the United States	46.5 (34-67)	7	3	10	2	0/4	2/6	NHL (10)	Pembrolizumab (10)
Armelle Lavole (38)	2021	Prospective cohort study	France	58 (44-71)	14	2	16	1	–	–	NSCLC (16)	Nivolumab (16)
Spano, Jean-Philippe (39)	2019	Retrospective study	France	62 (52.5-68.5)	18	5	23	2	–	–	NSCLC (21), metastatic melanoma (1), head and neck cancer (1)	Nivolumab (21), pembrolizumab (2)
Zer A (40)	2022	Prospective cohort study	Israeli	76 (61-87)	18	0	18	4	–	–	KS (18)	Nivolumab +ipilimumab (18)
Menghua Wu (41)	2023	Retrospective study	China	59.7 (41-75)	8	1	9	1	–	–	urothelial carcinoma (9)	Camrelizumab (9)

LC, lung cancer; HL, hodgkin lymphoma; NHL, non-Hodgkin lymphoma; NSCLC, non-small cell lung cancer; HCC, hepatocellular carcinoma; HNSCC, head and neck squamous cell carcinoma; SCLC, small cell lung cancer; KS, kaposi sarcoma; UC, urothelial carcinoma; RCC, renalcell carcinoma; NPC, nasopharyngeal carcinoma; BC, bladder cancer; GC, gastric cancer.

### 3.3 Incidence of severe irAEs (regardless of CD4+ T cell count)

**Figure 2:**
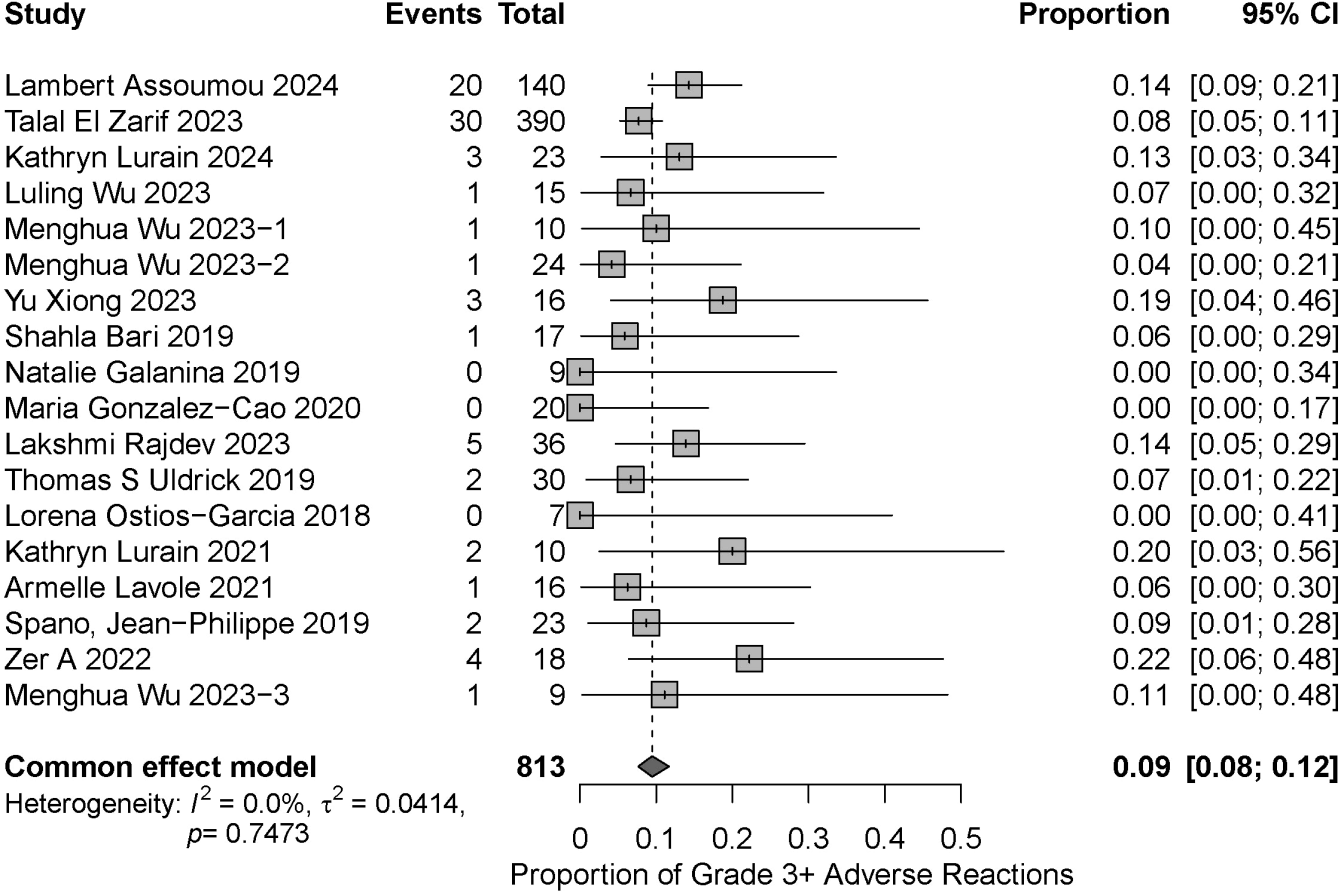
Forest plot and heterogeneity test for assessing the incidence of severe irAEs (regardless of CD4+ T cell count).

The incidence of grade 3 or higher irAEs in PLWH with cancer receiving ICIs was assessed in the 18 included studies. Heterogeneity testing revealed low statistical heterogeneity among the included studies (I² = 0%, P = 0.7473), which supports the consistency of the pooled analysis results. The pooled results are shown in **Figure 2**. Approximately 9% of patients experienced severe (grade 3 or higher) irAEs (95% CI: 0.08–0.12).

### 3.4 Sensitivity analysis and publication bias

**Figure 3:**
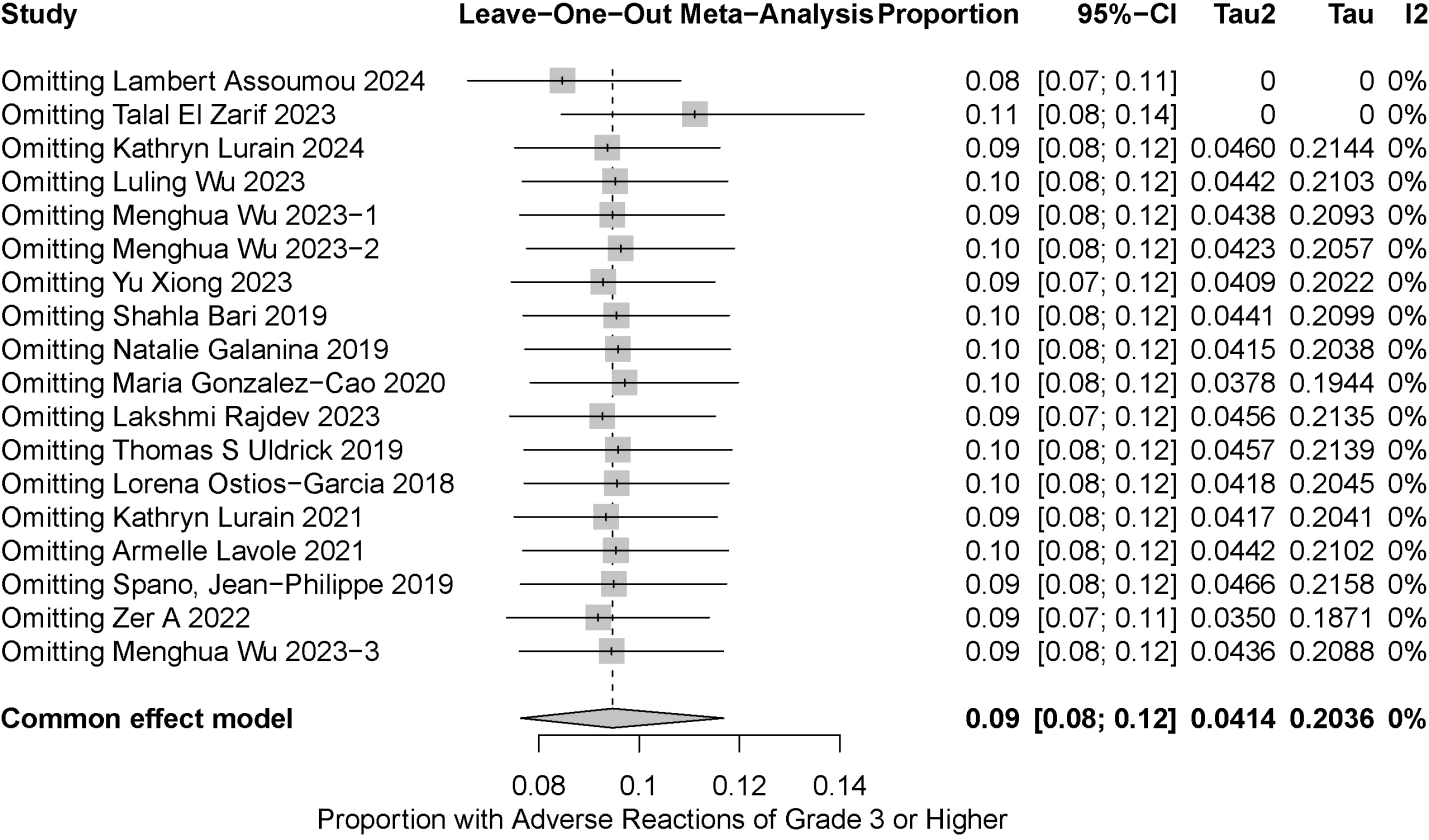
Sensitivity analysis to assess the incidence of severe irAEs (regardless of CD4+ T cell count).

**Figure 4:**
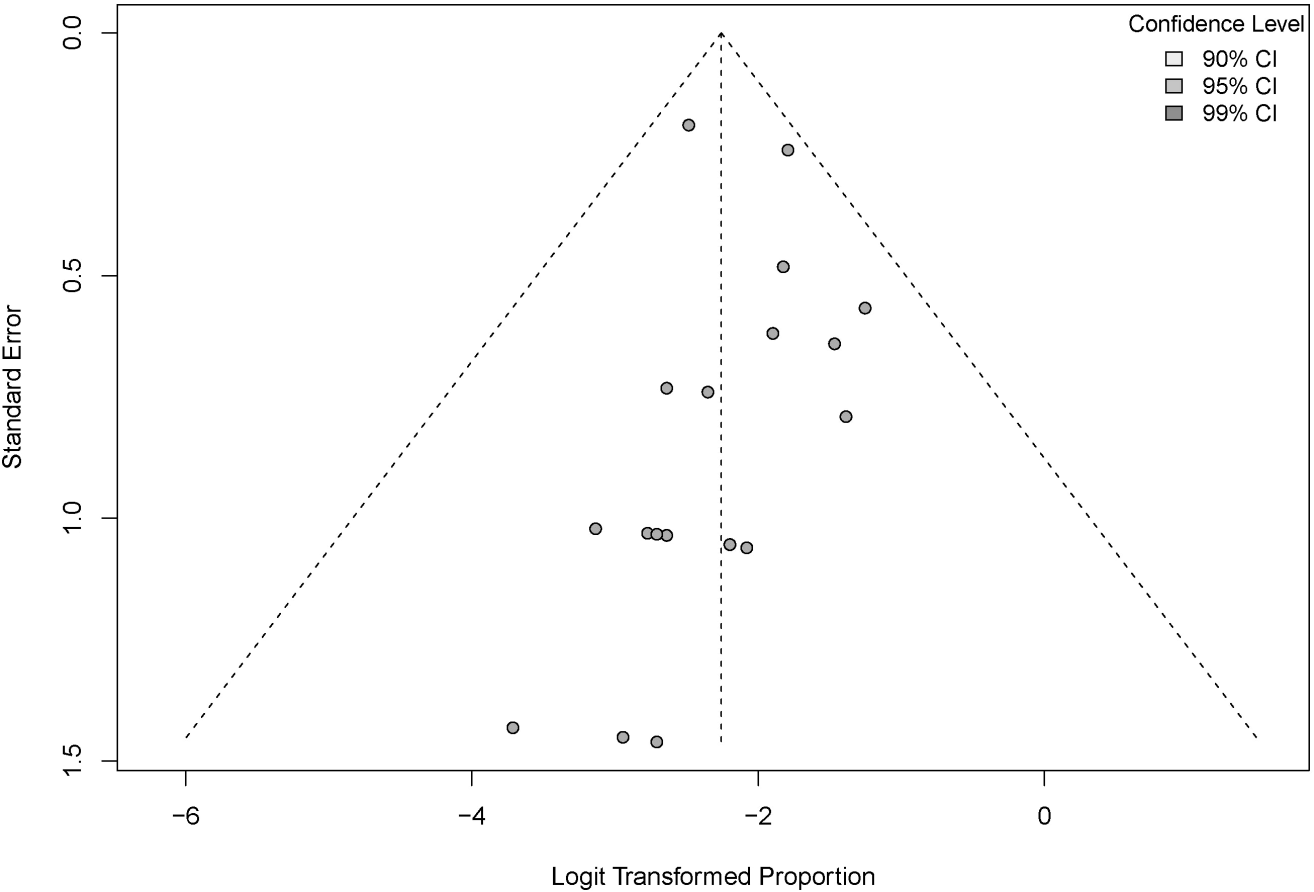
Funnel plot to assess the incidence of severe irAEs (regardless of CD4+ T cell count).

**Figure 5:**
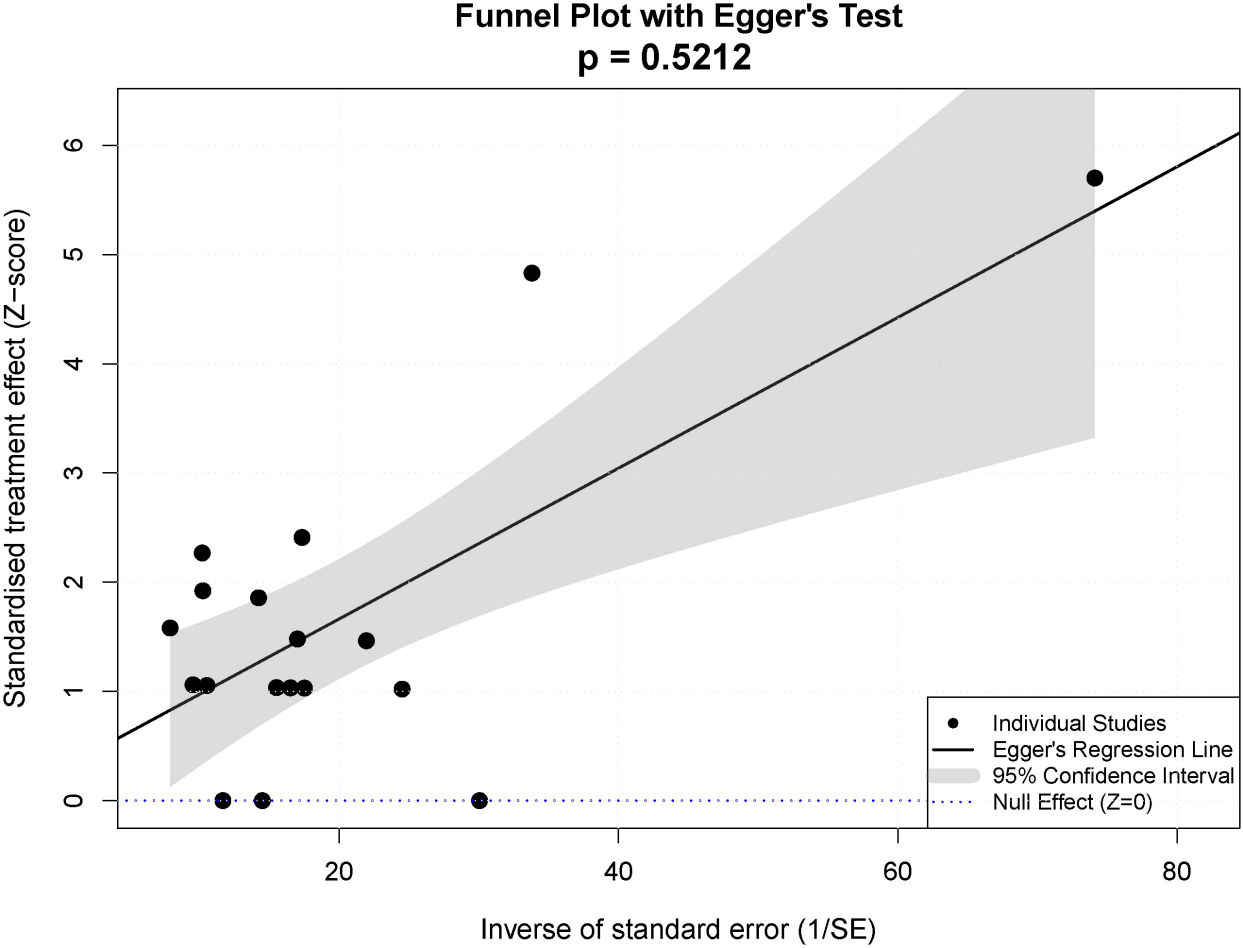
Egger’s test assesses the incidence of severe irAEs (regardless of CD4+ T cell count).

We conducted a sensitivity analysis using stepwise exclusion, and our results confirmed the stability and reliability of the pooled findings (**Figure 3**). Funnel plots and Egger’s test were used to comprehensively assess publication bias (**Figures 4**, **5**). Visual inspection of the funnel plot revealed asymmetry, suggesting potential publication bias. Egger’s test confirmed no significant asymmetry (p=0.5212). The results indicate the possibility of mild publication bias, but with a limited impact on the overall conclusions.

### 3.5 Incidence of severe irAEs (CD4+ T cell count < 200 vs. ≥ 200)

**Figure 6:**
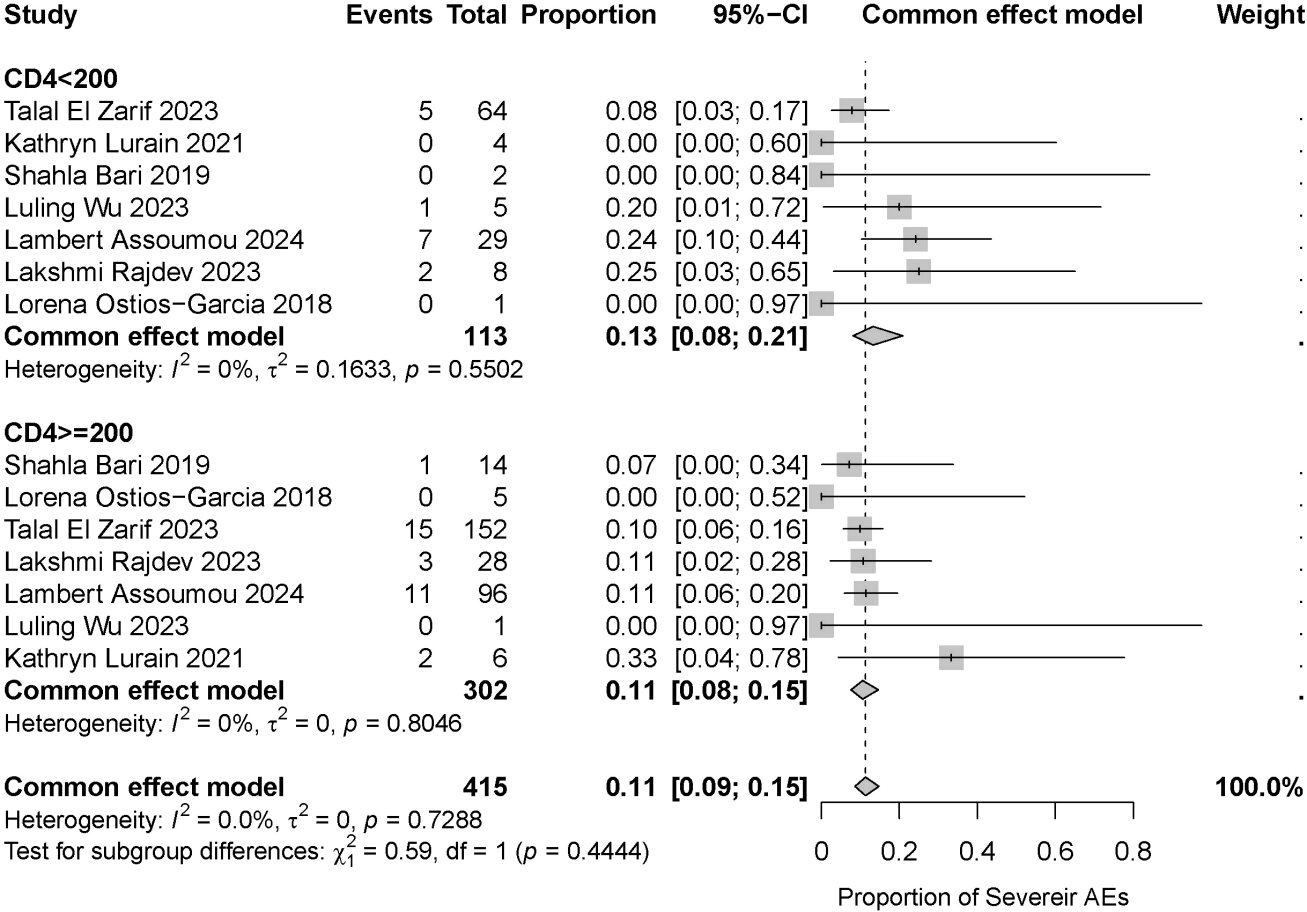
Incidence and heterogeneity of severe irAEs (CD4+ T cell count < 200 vs. ≥ 200).

We originally planned to conduct a subgroup analysis based on CD4+ T cell counts. However, this analysis was constrained by data availability, with only seven studies providing categorized reporting. Consequently, we conducted a meta-analysis of these seven studies. The pooled results are shown in **Figure 6**. The group with a CD4+ T cell count <200 included 113 patients. The incidence of severe irAEs was 13% (95% CI: 0.08–0.21). With low heterogeneity in this subgroup (I² = 0%, p = 0.5502), indicating consistent findings across the included studies. The group with a CD4+ T cell count ≥200 comprised 302 patients. The incidence of severe irAEs was 11% (95% CI: 0.08–0.15). Heterogeneity within this subgroup was low (I² = 0%, p = 0.8046), indicating consistent results across studies as well. The difference in the incidence of severe irAEs between the two groups was not statistically significant (p=0.4444). The overall pooled incidence was 11% (95% CI: 0.09–0.15), with no observed heterogeneity (I² = 0%, p = 0.7288). For further details on CD4+ T-cell counts and irAE incidence in the relevant studies, see **Table 1**.

### 3.6 Sensitivity and publication bias (CD4+ T cell count < 200 vs. CD4+ T cell count ≥ 200)

**Figure 7:**
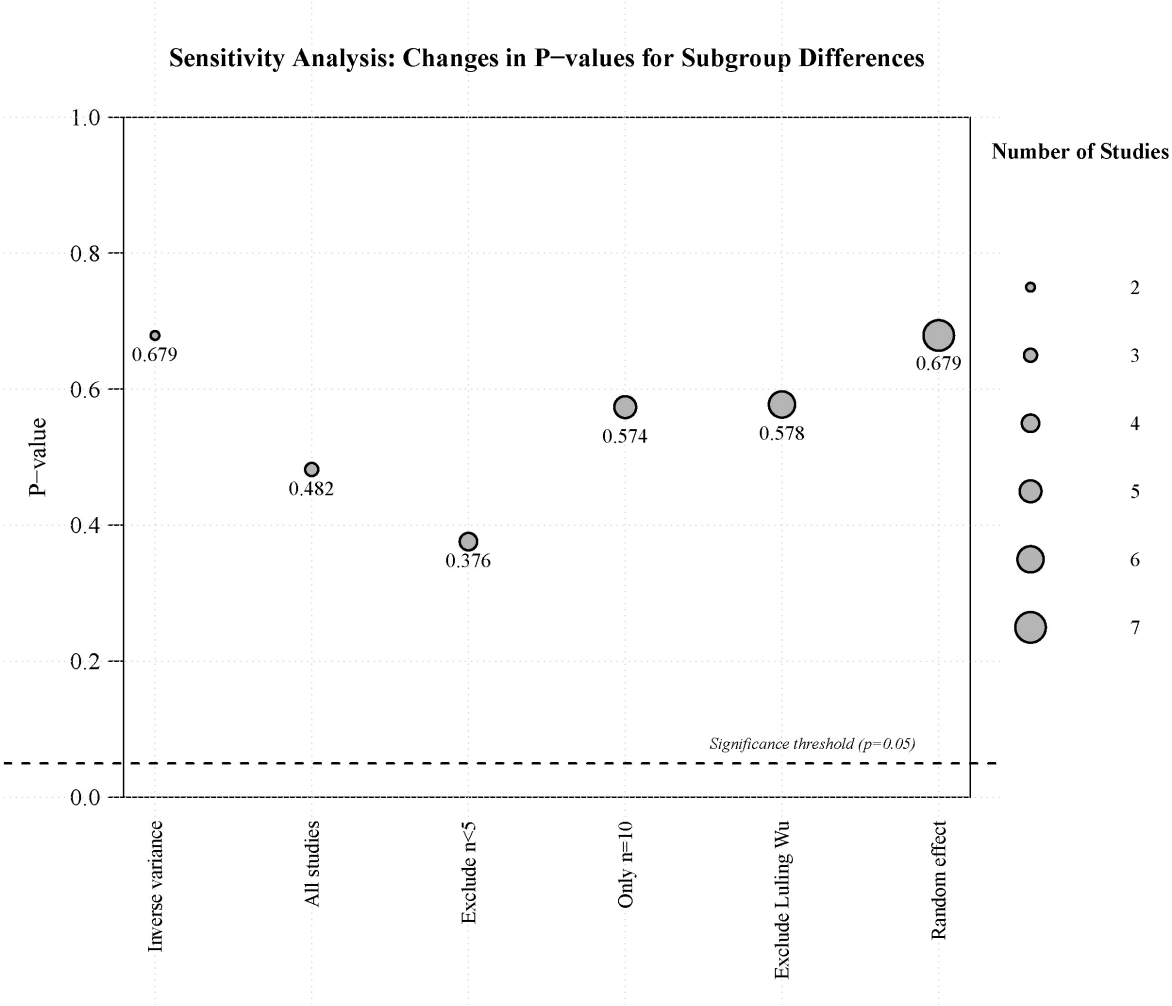
Sensitivity analysis to assess the incidence of severe irAEs (CD4+ T cell count < 200 vs. ≥ 200).

**Figure 8:**
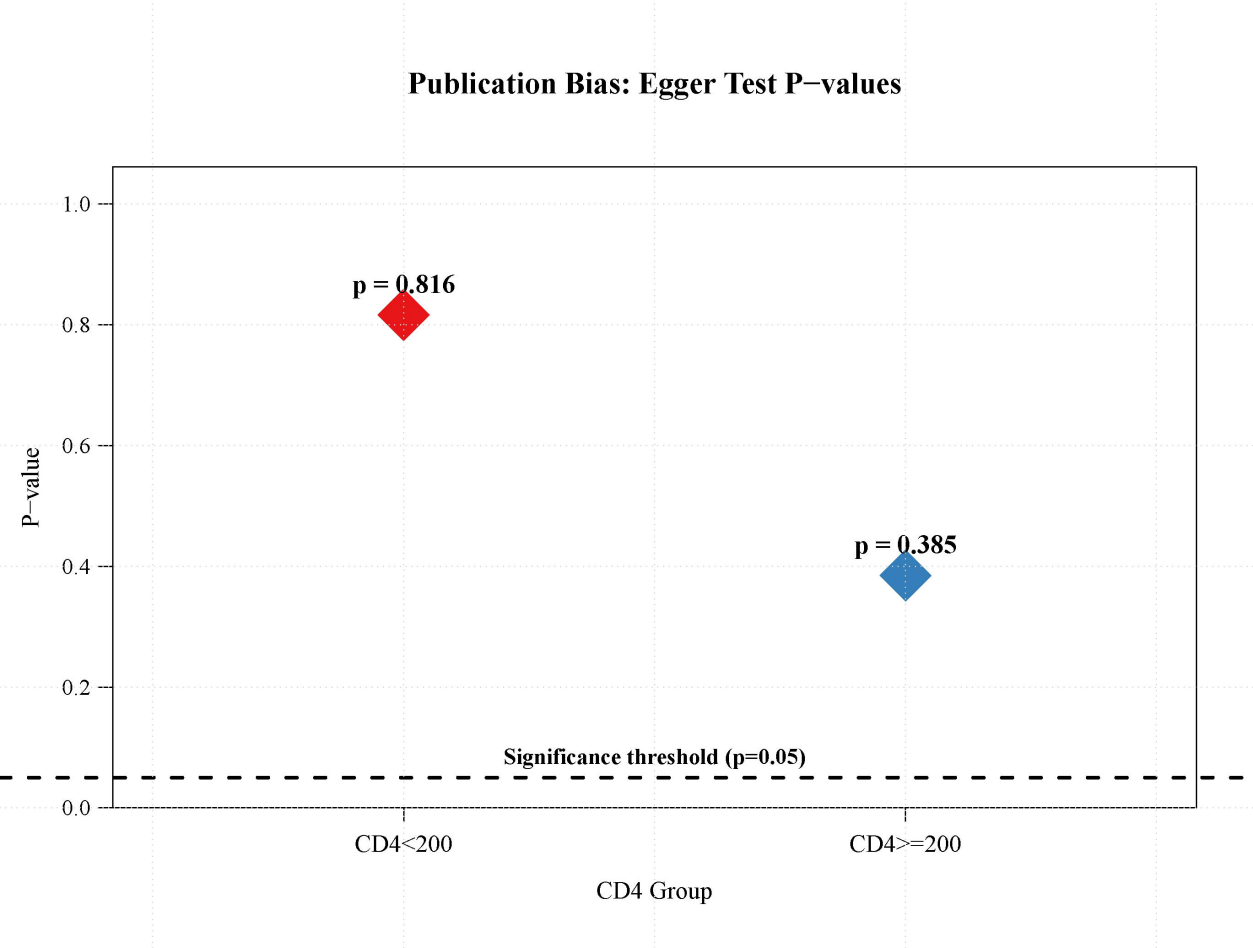
Egger’s test to assess the incidence of severe irAEs (CD4+ T cell count < 200 vs. ≥200).

**Figure 9:**
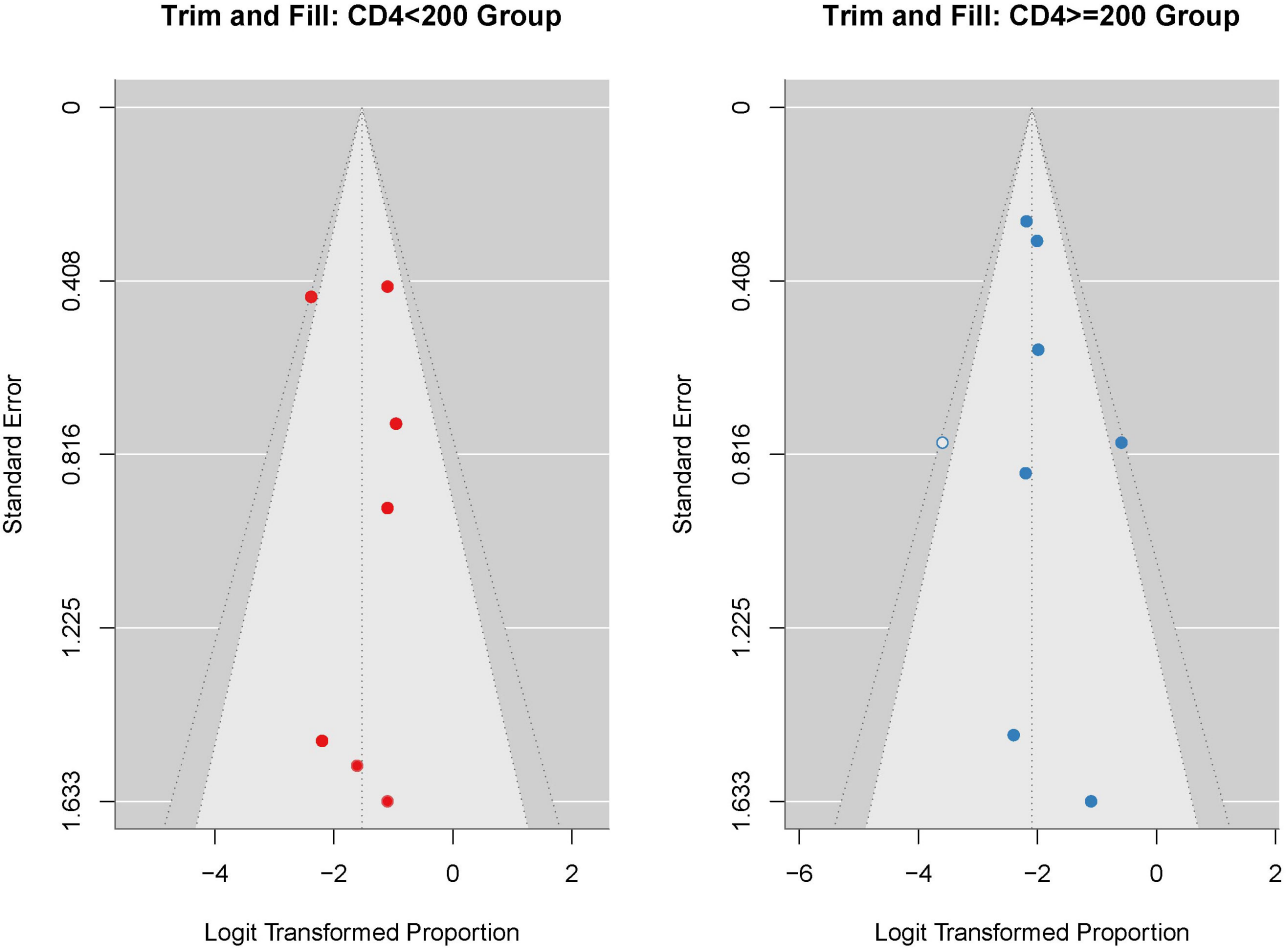
Funnel plot assessing the incidence of serious irAEs (CD4+ T cell count <200 vs. ≥200).

We conducted sensitivity analyses (**Figure 7**) to assess the robustness of subgroup differences across the included studies. Across all analytical scenarios, including the exclusion of studies with small sample sizes (n < 5), the inclusion of only studies with n = 10, and the application of random-effects models, the p-values for subgroup differences remained greater than 0.05. This consistency indicates that the observed subgroup differences were robust to variations in the inclusion criteria and analytical methods of the studies. To assess potential publication bias, we performed Egger regression tests for both CD4 subgroups (**Figure 8**). The results showed non-significant p-values for both subgroups (CD4 < 200: p = 0.816; CD4 ≥ 200: p = 0.385), indicating no apparent publication bias. Furthermore, we employed the trim-and-fill method to assess and impute potentially missing studies. The imputed funnel plot revealed symmetrical distributions across both groups, further supporting the absence of significant publication bias (**Figure 9**).

## 4 Discussion

Currently, ICIs have been widely used in cancer patients. Numerous studies have demonstrated the safety and efficacy of ICIs in HIV-uninfected cancer patients, with the incidence of their severe irAEs also remaining within manageable limits. Analyses of multiple clinical trial cohorts indicate that the incidence of severe irAEs is approximately 11–20% with anti-PD-1 agents and about 1–9% with anti-PD-L1 agents (11). A meta-analysis involving 12,808 cancer patients treated with anti-PD-1/PD-L1 monotherapy reported an overall severe irAE rate of 6.1%, with specific rates of 8.25% for nivolumab, 5.1% for pembrolizumab, and 5.28% for atezolizumab (12). Another meta-analysis of 8,730 patients receiving anti-PD-1/PD-L1 monotherapy showed severe irAE rates of 6.5% with anti-PD-1 therapy and 3.6% with anti-PD-L1 therapy (13).

This meta-analysis included 18 clinical studies involving 813 patients. Our objective was to determine the incidence of severe irAEs associated with ICI use in PLWH with cancer. The results of this review clearly indicate a 9% incidence of severe irAEs (95% CI: 0.08–0.12), irrespective of CD4+ T cell count. Although this incidence rate is higher than the rates reported in the two large meta-analyses of HIV-uninfected cancer patients mentioned in the preceding paragraph, it does not represent a difference of an order of magnitude, and the numerical ranges are relatively close. Furthermore, this rate falls within the range of severe irAE rates (1%–9% and 11%–20%) reported in multiple clinical trials involving HIV-uninfected cancer patients. Therefore, taken together, the evidence suggests that ICIs have an acceptable safety profile in cancer patients with chronic HIV infection. In addition, we extracted data from seven studies to further stratify CD4 + T cell counts. The results showed that the difference in the incidence of severe irAEs was not statistically significant between the CD4+ T cell count <200 and ≥200 groups (p=0.4444). However, the wider confidence interval in the CD4+ T cell count<200 subgroup reflects both the uncertainty stemming from its smaller sample size and the inherent clinical heterogeneity within this population. In clinical practice, patients with a CD4+ T-cell count <200 still require closer monitoring. This finding differs from the trend observed in some HIV-uninfected cancer patients, where a ‘higher CD4+ T cell count is associated with a higher risk of irAEs’ (14). This discrepancy may originate from the inherent, persistent immune dysregulation state in PLWH, which continues even after cART. In this context, the numerical value of the peripheral blood CD4+ T cell count alone may no longer fully represent the functional integrity and reactive potential of their immune system, thereby diminishing its efficacy as a predictive biomarker for irAEs. This highlights the need to identify more precise immune biomarkers than the CD4+ T cell count within this special population of PLWH to predict both treatment response and toxicity. Concurrently, low CD4 counts themselves represent a significant risk factor for non-immune events such as infections among PLWH. We must clearly distinguish these from irAEs and strictly adhere to the inclusion criteria for irAEs when collecting data.

Under normal conditions, regulatory molecules on the surface of T cells (CTLA-4 and PD-1) bind to ligands (B7 family and PD-L1) to inhibit the overactivation of T cells and maintain an autoimmune state (15). In contrast, tumor cells bind to T cells through the high expression of ligands, such as PD-L1 (16). Tumor cells inhibit the antitumor activity of T cells by transmitting inhibitory signals, thus facilitating immune escape (16). In contrast, ICIs (including CTLA-4 and PD-1/PD-L1 inhibitors) restore antitumor immunity by blocking T cell inhibitory signals and reactivating T cell killing of tumors (17). In PLWH, the virus binds to receptors on the surface of CD4^+^ T cells via its envelope glycoprotein gp120, reverse transcribes its RNA into DNA, and integrates this DNA into the host genome (18). During the acute infection stage, HIV replicates extensively within lymphoid tissues, leading to a sharp decline in CD4+ T cell counts while simultaneously triggering a robust immune response (19). The high mutation rate of HIV results in the continuous generation of viral variants capable of evading specific immune responses. Furthermore, the virus can establish latency within resting T cells, macrophages, or dendritic cells, forming a persistent viral reservoir that is difficult to eradicate. During the chronic phase of HIV infection, persistent low-level viral replication and the release of viral proteins, combined with microbial translocation resulting from the breakdown of the gut mucosal barrier, collectively drive non-resolving systemic immune activation and chronic inflammation (20). This fosters an environment rich in pro-inflammatory cytokines such as IL-1 and IL-6, which not only accelerates immune dysfunction but also creates favorable conditions for the virus’s persistence (21). Concurrently, virus-specific CD8+ T cells enter a state of functional exhaustion, characterized by the high expression of inhibitory receptors like PD-1 (22). Although this upregulation of inhibitory pathways temporarily prevents excessive immune activation and associated tissue damage—thereby sustaining host survival—it directly paralyzes effective immune effector functions, inadvertently shielding the viral reservoir from clearance. Ultimately, these interconnected processes establish a dysfunctional “balance” between HIV and its host. The immune system, by upregulating inhibitory pathways, tenuously contains the damage inflicted by both the virus and the ensuing chronic inflammation. However, this comes at the cost of the progressive erosion of its own functional capacity and a comprehensive decline in immune surveillance. Therefore, in PLWH with cancer, ICIs, on the one hand, deregulate T cell suppression and process tumor cells. On the other hand, they also restore the function of some HIV-specific CD8+ T cells and enhance their ability to clear cells with latent HIV infection. Thus, they kill two birds with one stone. However, ICIs are a double-edged sword. This immune activation may disrupt the equilibrium between HIV and the host immune system, leading to immune overactivation, which can trigger immune reconstitution inflammatory syndrome (IRIS) (23). Therefore, in the presence of this potential safety risk, PLWH are usually excluded from pivotal clinical trials for most ICIs. In contrast, according to the results of our meta-analysis, the use of ICIs in PLWH with cancer did not result in a significantly higher incidence of serious irAEs.

The meta-analysis of the incidence of serious irAEs (irrespective of CD4+ T cell counts) showed no significant heterogeneity (I² = 0%). To ensure the robustness of our findings, we performed sensitivity analyses, which confirmed the stability of the pooled results. Meta-analyses of severe irAE incidence stratified by CD4+ T cell count (<200 vs. ≥200 cells/μL) showed excellent homogeneity within both the CD4+ T cell count <200 group (I² = 0%) and the CD4+ T cell count ≥200 group (I² = 0%), indicating highly reliable estimates. Although no statistically significant differences were observed between the two groups, the wide confidence interval in the CD4+ T cell count <200 group still indicates the need for close monitoring. Patients with greater immunosuppression may require greater vigilance for irAEs. We also demonstrated the robustness of the finding that there was no statistically significant difference between the two groups by performing a series of sensitivity analyses for different scenarios.

We assessed publication bias in the incidence of serious irAEs (regardless of the CD4+ T cell count). We used both funnel plots and Egger’s test, and the funnel plots showed mild asymmetry, consistent with Egger’s test (p > 0.05). These results indicated a mild risk of publication bias. In combination with the sensitivity results, we concluded that the meta-analysis results were generally reliable. This may be related to the fact that positive results are more likely to be published and that studies with a high incidence of adverse reactions deserve more attention. To assess the incidence of serious irAEs (CD4+ T cell count <200 vs.≥200), we used the Egger test and the trim-and-fill method. The results showed no significant publication bias. This enhances the credibility and replicability of the findings.

The studies included in this review primarily aimed to assess the safety of ICIs in cancer patients with HIV. Consequently, there was significant heterogeneity across studies in the reporting of efficacy endpoints—such as objective response rate (ORR) and progression-free survival (PFS)—as well as in their definitions and follow-up durations, precluding a quantitative pooled analysis. Nevertheless, several studies reported encouraging signals of antitumor activity. For example, the study by Talal El Zarif et al. directly compared ICI outcomes between HIV-infected and HIV-uninfected patients NSCLC and found similar overall survival (OS), PFS, and ORR between the two groups; ORRs for other cancer types also followed trends consistent with those observed in HIV-uninfected cancer patients (24). In the study by Kathryn Lurain et al., ICIs achieved an ORR of 83% in HL, with a median duration of response (DOR) of 19.7 months and a median PFS of 21.2 months—results that fall within the range reported in prior trials of anti-PD-1 monotherapy (25). Additionally, a retrospective analysis by Luling Wu et al. of 15 PLWH with advanced cancer treated with PD-1 inhibitors showed a disease control rate (DCR) of 53.3% (26). Although these data are preliminary and fragmented, they collectively indicate that ICIs are not ineffective in PLWH with cancer and hold clear potential for clinical benefit.

However, our study had some limitations. First, our search strategy was designed *a priori* to broadly capture the class of “immune checkpoint inhibitors.” As insightfully suggested during peer review, incorporating specific drug names and proximity operators in future systematic reviews could further enhance search sensitivity and comprehensiveness. Second, the inclusion criteria for recruiting PLWH in the original trials we incorporated varied significantly and were generally broad (often requiring only “HIV infection” and “confirmed malignancy”). Owing to the lack of unified, more refined conditions (such as a lower threshold for CD4+ T cell counts, virologic suppression status, or specific antiretroviral therapy regimens), we were unable to perform homogeneous subgroup analyses based on patients’ immunological status. This may have introduced heterogeneity in the baseline immune function of the study population, thereby limiting our ability to assess the risk for specific subgroups more precisely. Future large-scale studies should conduct more detailed stratified analyses of patient populations, treatment regimens, and outcome definitions to generate more precise conclusions. Third, although data on severe irAEs were extracted in strict accordance with our predefined criteria, residual heterogeneity due to variations in reporting detail across original studies may still persist. Some studies may not have comprehensively reported the details used to judge the immune-relatedness of all adverse events. Therefore, the pooled incidence rates presented in this report should be interpreted as estimates based on the best currently available evidence, processed under our unified rules. The adoption of standardized adverse event reporting practices in future prospective studies is of paramount importance, especially for special populations with complex comorbidities such as PLWH. Fourth, only seven studies were included in the analysis comparing CD4+ T cell counts <200 vs. ≥200, and a significant proportion of these were small-sample studies. The number and sample size of the included studies limited our conclusions. Further high-quality studies are required to validate these findings. Moreover, the sample size in the group with CD4+ T cell count <200 was only one-third that of the other subgroups, potentially affecting the statistical power. Therefore, future studies should focus on ICIs in this specific population with CD4+ T cell count < 200. Concurrently, more research is needed regarding the correlation between HIV-specific factors (such as CD4 count and viral load) and the risk of irAEs. Fifth, this study is a systematic review and meta-analysis focused on safety. Due to high heterogeneity in reporting efficacy endpoints (such as ORR and PFS) across the included original studies, we were unable to perform a quantitative pooled analysis. Consequently, this study cannot provide a summary estimate of the efficacy of ICIs. Although we summarize preliminary, encouraging signals of antitumor activity in the discussion section, a comprehensive risk-benefit profile assessment requires an integrated analysis of efficacy and safety data. This critical evaluation awaits future prospectively designed clinical trials specifically designed to simultaneously and rigorously assess both efficacy and safety outcomes in this population. Concurrently, all studies included in this review were observational, with no randomized controlled trials. It is hoped that future randomized controlled trials of ICIs will include PLWH, enabling more precise comparisons with HIV-uninfected cancer patients. Implementing stratified randomization and robust safety monitoring protocols will provide higher-level evidence supporting immunotherapy in PLWH with cancer. Furthermore, future studies should incorporate longitudinal monitoring of HIV disease status—such as CD4+ T cell counts and HIV viral load—to elucidate the interactions between ICI therapy and HIV infection, thereby providing a foundation for comprehensive management of PLWH with cancer.

## 5 Conclusion

Preliminary data from this study show that the pooled incidence of severe irAEs induced by ICIs is approximately 9% in ART-treated and clinically stable PLWH with cancer. The rate is relatively close to the spectrum of irAE incidence observed with ICI therapy in HIV-uninfected cancer patients. Furthermore, subgroup analysis based on CD4+ T cell count revealed no statistically significant difference in the incidence of severe irAEs between the groups. Therefore, current evidence does not indicate that baseline CD4+ T cell count is an absolute contraindication to ICI therapy in PLWH with cancer. Taken together, these findings provide preliminary support for an acceptable safety profile of ICIs in PLWH with cancer. Combined with observed preliminary antitumor activity, the results justify further prospective studies. However, the wider confidence interval for the incidence in the subgroup with CD4+ T cell count <200 suggests greater uncertainty in this population, warranting closer monitoring in clinical practice.
