# Supplementary material for: Incidence of severe immune-related adverse reactions in patients with HIV and cancer receiving immune checkpoint inhibitors: a systematic review and meta-analysis
Source: Front Oncol. 2026 Mar 17;16:1741760. doi: 10.3389/fonc.2026.1741760 (PMC13035766; doi:10.3389/fonc.2026.1741760)
Supplement: Supplementary file 1 [file DataSheet1.pdf]

Additional File 1 Search strategy

Date 2025.7.13

Pubmed

https://pubmed.ncbi.nlm.nih.gov/

("Tumors" OR "Neoplasia" OR "Neoplasias" OR "Neoplasm" OR "Tumor" OR " Cancer" OR "Cancers" OR "Malignant Neoplasm" OR "Malignancy" OR" Malignancies" OR "Malignant Neoplasms" OR "Neoplasm, Malignant" OR "Neoplasms, Malignant") AND ("HIV" OR "AIDS" OR "Human Immunodeficiency Virus" OR " Immunodeficiency Virus, Human" OR " Immunodeficiency Viruses, Human" OR "Virus, Human Immunodeficiency" OR "Viruses, Human Immunodeficiency" OR "Human Immunodeficiency Viruses" OR "HTLV-III" OR "Human T Cell Lymphotropic Virus Type III" OR "Human T-Cell Lymphotropic Virus Type III" OR " Human T-Cell Leukemia Virus Type III" OR "Human T Cell Leukemia Virus Type III" OR "LAV-HTLV-III" OR " Lymphadenopathy-Associated Virus" OR "Lymphadenopathy Associated Virus" OR "Lymphadenopathy-Associated Viruses" OR "Viruses, Lymphadenopathy-Associated" OR "Virus, Lymphadenopathy-Associated" OR "Human T Lymphotropic Virus Type III" OR "Human T-Lymphotropic Virus Type III" OR "AIDS Virus" OR " AIDS Viruses" OR "Virus, AIDS" OR "Viruses, AIDS" OR "Acquired Immune Deficiency Syndrome Virus" OR "Acquired Immunodeficiency Syndrome Virus") AND ("Immune Checkpoint Inhibitors" OR "Immune Checkpoint Inhibitor" OR "Checkpoint Inhibitors" OR "Checkpoint Inhibitor" OR "Immune Checkpoint Blockers" OR "Checkpoint Blockers" OR "Immune Checkpoint Blockade" OR "Checkpoint Blockade" OR "Immune Checkpoint Inhibition" OR "Checkpoint Inhibition" OR "PD-L1 Inhibitors" OR "PD L1 Inhibitors" OR "PD-L1 Inhibitor" OR "PD L1 Inhibitor" OR "Programmed Death-Ligand 1 Inhibitors" OR "Programmed Death Ligand 1 Inhibitors" OR "PD-1/PD-L1 Blockade" OR "Blockade PD-1/PD-L1" OR "PD 1 PD L1 Blockade" OR "PD-1 Inhibitors" OR "PD 1 Inhibitors" OR "PD-1 Inhibitor" OR "PD-1 Inhibitor" OR "PD 1 Inhibitor" OR "Programmed Cell Death Protein 1 Inhibitor" OR "Programmed Cell Death Protein 1 Inhibitors")

Web of science

https://webofscience.clarivate.cn/wos/woscc/smart-search

nce

(TS=("Tumors") OR TS=("Neoplasia") OR TS=("Neoplasias") OR TS=("Neoplasm") OR TS=("Tumor") OR TS=(" Cancer") OR TS=("Cancers") OR TS=("Malignant Neoplasm") OR TS=("Malignancy") OR TS=("Malignancies") OR TS=("Malignant Neoplasms") OR TS=("Neoplasm, Malignant") OR TS=("Neoplasms, Malignant")) AND (TS=("HIV") OR TS=("AIDS") OR TS=("Human Immunodeficiency Virus") OR TS=(" Immunodeficiency Virus, Human") OR TS=("Immunodeficiency Viruses, Human") OR TS=(" Virus, Human Immunodeficiency") OR TS=("Viruses, Human Immunodeficiency") OR TS=(" Human Immunodeficiency Viruses") OR TS=(" HTLV-III") OR TS=(" Human T Cell Lymphotropic Virus Type III") OR TS=(" Human T-Cell Lymphotropic Virus Type III") OR TS=(" Human T-Cell Leukemia Virus Type III") OR TS=(" Human T Cell Leukemia Virus Type III") OR TS=(" LAV-HTLV-III") OR TS=(" Lymphadenopathy-Associated Virus") OR TS=(" Lymphadenopathy Associated Virus") OR TS=(" Lymphadenopathy-Associated Viruses") OR TS=("Viruses, Lymphadenopathy-Associated") OR TS=("Virus, Lymphadenopathy-Associated") OR TS=("Human T Lymphotropic Virus Type III") OR TS=("Human T-Lymphotropic Virus Type III") OR TS=("AIDS Virus") OR TS=("AIDS Viruses") OR TS=("Virus, AIDS") OR TS=("Viruses, AIDS") OR TS=(" Acquired Immune Deficiency Syndrome Virus") OR TS=("Acquired Immunodeficiency Syndrome Virus"))AND (TS=("Immune Checkpoint Inhibitors") OR TS=("Immune Checkpoint Inhibitor") OR TS=("Checkpoint Inhibitors") OR TS=("Checkpoint Inhibitor") OR TS=("Immune Checkpoint Blockers") OR TS=("Checkpoint Blockers") OR TS=("Immune Checkpoint Blockade") OR TS=("Checkpoint Blockade") OR TS=("Immune Checkpoint Inhibition") OR TS=("Checkpoint Inhibition") OR TS=("PD-L1 Inhibitors") OR TS=("PD L1 Inhibitors") OR TS=("PD-L1 Inhibitor") OR TS=("PD L1 Inhibitor") OR TS=("Programmed Death-Ligand 1 Inhibitors") OR TS=("Programmed Death Ligand 1 Inhibitors") OR TS=("PD-1/PD-L1 Blockade") OR TS=("Blockade PD-1/PD-L1") OR TS=("PD 1 PD L1 Blockade") OR TS=("PD-1 Inhibitors") OR TS=("PD 1 Inhibitors") OR TS=("PD-1 Inhibitor") OR TS=("PD-1 Inhibitor") OR TS=("PD 1 Inhibitor") OR TS=("Programmed Cell Death Protein 1 Inhibitor") OR TS=("Programmed Cell Death Protein 1 Inhibitors"))

Embase

https://www.embase.com/landing?status=grey

(tumors:ti,ab,kw OR neoplasia:ti,ab,kw OR neoplasias:ti,ab,kw OR neoplasm:ti,ab,kw OR tumor:ti,ab,kw OR cancer:ti,ab,kw OR cancers:ti,ab,kw OR 'malignant neoplasm':ti,ab,kw OR malignancy:ti,ab,kw OR malignancies:ti,ab,kw OR 'malignant neoplasms':ti,ab,kw OR 'neoplasm,

malignant':ti,ab,kw OR 'neoplasms, malignant':ti,ab,kw) AND (hiv:ti,ab,kw OR aids:ti,ab,kw OR 'human immunodeficiency virus':ti,ab,kw OR 'immunodeficiency virus, human':ti,ab,kw OR 'immunodeficiency viruses, human':ti,ab,kw OR 'virus, human immunodeficiency':ti,ab,kw OR 'viruses, human immunodeficiency':ti,ab,kw OR 'human immunodeficiency viruses':ti,ab,kw OR htlv-iii:ti,ab,kw OR 'human t cell lymphotropic virus type iii':ti,ab,kw OR 'human t-cell lymphotropic virus type iii':ti,ab,kw OR 'human t-cell leukemia virus type iii':ti,ab,kw OR 'human t cell leukemia virus type iii':ti,ab,kw OR 'lav-htlv-iii':ti,ab,kw OR 'lymphadenopathy-associated virus':ti,ab,kw OR 'lymphadenopathy associated virus':ti,ab,kw OR 'lymphadenopathy-associated viruses':ti,ab,kw OR 'viruses, lymphadenopathy-associated':ti,ab,kw OR 'virus, lymphadenopathy-associated':ti,ab,kw OR 'human t lymphotropic virus type iii':ti,ab,kw OR 'human t-lymphotropic virus type iii':ti,ab,kw OR 'aids virus':ti,ab,kw OR 'aids viruses':ti,ab,kw OR 'virus, aids':ti,ab,kw OR 'viruses, aids':ti,ab,kw OR 'acquired immune deficiency syndrome virus':ti,ab,kw OR 'acquired immunodeficiency syndrome virus':ti,ab,kw) AND ('immune checkpoint inhibitors':ti,ab,kw OR 'immune checkpoint inhibitor':ti,ab,kw OR 'checkpoint inhibitors':ti,ab,kw OR 'checkpoint inhibitor':ti,ab,kw OR 'immune checkpoint blockers':ti,ab,kw OR 'checkpoint blockers':ti,ab,kw OR 'immune checkpoint blockade':ti,ab,kw OR 'checkpoint blockade':ti,ab,kw OR 'immune checkpoint inhibition':ti,ab,kw OR 'checkpoint inhibition':ti,ab,kw OR 'pd-l1 inhibitors':ti,ab,kw OR 'pd l1 inhibitors':ti,ab,kw OR 'pd-l1 inhibitor':ti,ab,kw OR 'pd l1 inhibitor':ti,ab,kw OR 'programmed death-ligand 1 inhibitors':ti,ab,kw OR 'programmed death ligand 1 inhibitors':ti,ab,kw OR 'pd-1/pd-l1 blockade':ti,ab,kw OR 'blockade pd-1/pd-l1':ti,ab,kw OR 'pd 1 pd l1 blockade':ti,ab,kw OR 'pd-1 inhibitors':ti,ab,kw OR 'pd 1 inhibitors':ti,ab,kw OR 'pd-1 inhibitor':ti,ab,kw OR 'pd 1 inhibitor':ti,ab,kw OR 'programmed cell death protein 1 inhibitor':ti,ab,kw OR 'programmed cell death protein 1 inhibitors':ti,ab,kw)
