# Supplementary material for: Incidence of severe immune-related adverse reactions in patients with HIV and cancer receiving immune checkpoint inhibitors: a systematic review and meta-analysis
Source: Front Oncol. 2026 Mar 17;16:1741760. doi: 10.3389/fonc.2026.1741760 (PMC13035766; doi:10.3389/fonc.2026.1741760)
Supplement: Supplementary file 2 [file DataSheet2.pdf]

Study	Total score	Selection				Comparability		Outcome		
		Representative-ness	Selection of non-exposure	Ascertainment of exposure	Outcome not present at start	Comparability on most important factors	Comparability on other risk factors	Assessment of outcome	Long enough follow-up	Adequacy (completeness) of follow-up
Lambert Assoumou 2024	8	☆	N/A	☆	☆	☆	☆	☆	☆	☆
Talal El Zarif 2023	7	☆	N/A	☆	☆	☆		☆	☆	☆
Kathryn Lurain 2024	6	☆	N/A	☆	☆	–	–	☆	☆	☆
Luling Wu 2023	8	☆	N/A	☆	☆	☆	☆	☆	☆	☆
Menghua Wu 2023-1	6	☆	N/A	☆	☆	–	–	☆	☆	☆
Menghua Wu 2023-2	6	☆	N/A	☆	☆	–	–	☆	☆	☆
Yu Xiong 2023	6	☆	N/A	☆	☆	–	–	☆	☆	☆
Shahla Bari 2019	8	☆	N/A	☆	☆	☆	☆	☆	☆	☆
Natalie Galanina 2019	6	☆	N/A	☆	☆	–	–	☆	☆	☆
Maria Gonzalez-Cao 2020	6	☆	N/A	☆	☆	–	–	☆	☆	☆
Lakshmi Rajdev 2023	7	☆	N/A	☆	☆	☆	–	☆	☆	☆
Thomas S Uldrick 2019	6	☆	N/A	☆	☆	–	–	☆	☆	☆
Lorena Ostios-Garcia 2018	8	☆	N/A	☆	☆	☆	☆	☆	☆	☆
Kathryn Lurain 2021	8	☆	N/A	☆	☆	☆	☆	☆	☆	☆
Armelle Lavole 2021	6	☆	N/A	☆	☆	–	–	☆	☆	☆
Spano, Jean-Philippe 2019	6	☆	N/A	☆	☆	–	–	☆	☆	☆
Zer A 2022	6	☆	N/A	☆	☆	–	–	☆	☆	☆
Menghua Wu 2023-3	7	☆	N/A	☆	☆	☆	–	☆	☆	☆
